# Phosphorus Supply Under Micro-Nano Bubble Water Drip Irrigation Enhances Maize Yield and Phosphorus Use Efficiency

**DOI:** 10.3390/plants13213046

**Published:** 2024-10-30

**Authors:** Qingyong Bian, Zhiduo Dong, Yupeng Zhao, Yaozu Feng, Yanbo Fu, Zhiguo Wang, Jingquan Zhu

**Affiliations:** 1 Institute of Soil Fertilizer, Agricultural Water Saving, Xinjiang Academy of Agricultural Sciences, Urumqi 830091, China; 18116873795@126.com (Q.B.); dzd1281228561@163.com (Z.D.); 17797814646@163.com (Y.Z.); fuyanbo@xaas.ac.cn (Y.F.); 2 National Soil Quality Aksu Observation Experimental Station, Aksu 843000, China; ayg13779155706@163.com; 3 Scientific and Technological Achievement Transformation Center, Xinjiang Academy of Agricultural Sciences, Urumqi 830091, China

**Keywords:** *Zea mays* L., soil enzyme activity, grow, P uptake

## Abstract

This study aimed to explore the combined effects of micro-nano bubble water drip irrigation and different phosphorus (P) application rates (P0: 0 kg·hm^−2^; P1: 86 kg·hm^−2^; P2: 172 kg·hm^−2^; P3: 258 kg·hm^−2^) on maize growth, soil phosphorus dynamics, and phosphorus use efficiency to optimize irrigation and P fertilizer use efficiency. Through a field column experiment, the impact of micro-nano bubble water drip irrigation on maize plant height, stem diameter, leaf SPAD values, biomass, and yield was evaluated. The results showed that (1) irrigation methods significantly affected maize growth indicators such as plant height, stem diameter, and root dry weight. Micro-nano bubble water drip irrigation consistently promoted growth during all growth stages, especially under higher P application. (2) P application significantly increased the dry weight and P concentration in maize roots, stems, leaves, ears, and grains. Under micro-nano bubble water drip irrigation, the P concentrations in roots and grains increased by 59.28% to 92.59%. (3) Micro-nano bubble water drip irrigation significantly enhanced P uptake efficiency, partial factor productivity of P, and agronomic P use efficiency. Particularly under P1 and P2 treatments, the increases were 134.91% and 45.42%, respectively. Although the effect on apparent P recovery efficiency was relatively small, micro-nano bubble water drip irrigation still improved P utilization under moderate P levels. (4) Structural equation modeling indicated that P supply under micro-nano bubble water drip irrigation primarily regulated alkaline protease and alkaline phosphatase, enhancing soil P availability, which in turn promoted maize P accumulation and increased yield. In conclusion, this study demonstrated that the combination of micro-nano bubble water drip irrigation and appropriate P application can effectively promote maize growth and nutrient utilization, providing a theoretical basis for optimizing irrigation and fertilization strategies in maize production.

## 1. Introduction

Maize (*Zea mays* L.) is one of the most widely cultivated crops globally [1]. China, as the second-largest producer, contributes 261 million t annually, accounting for 23% of the global maize output [2]. In Xinjiang, a critical agricultural production region and major grain base, the annual maize yield reaches 9.28 million t, representing 59% of the region’s total grain production [3]. However, agriculture in Xinjiang faces significant challenges, including soil salinization, poor fertility, and desertification [4]. These issues have negatively impacted soil health and restricted nutrient availability, particularly phosphorus [5,6].

Phosphorus (P) is an essential nutrient for plant growth [7,8]. Yet, in Xinjiang, the predominance of alkaline soils, characterized by high P fixation and low bioavailability [9,10,11], severely limits maize’s ability to absorb this vital element, consequently restricting crop productivity [6]. Therefore, effective management practices are urgently needed to enhance P availability [6,7,12]. To address future food security challenges, developing efficient strategies to improve phosphorus utilization is critical [13].

Micro-nano bubble technology, by injecting oxygen and nutrient-rich nanobubbles into irrigation water, represents an innovative approach to enhance soil oxygenation, nutrient cycling, and plant health. This technology can significantly improve water quality by injecting oxygen and ionic nutrients into the soil solution [14,15,16], which not only raises soil oxygen concentration but also alters the composition and diversity of the soil bacterial community, thereby regulating its structure and functions [16,17]. By enhancing the cation exchange capacity and hydrophilicity of the soil matrix, micro-nano bubble irrigation accelerates organic matter decomposition, achieving rates two to six times faster than under anaerobic conditions [18]. Soil oxygen levels also critically impact nitrification and denitrification processes [16], with nitrification rates declining by six to nine times when oxygen partial pressure falls from 20.4 kPa to 0.35 kPa, a change that is crucial for chemical reactions and element cycling in the soil [19]. Furthermore, micro-nano bubble irrigation supports aerobic respiration in plant roots, enabling them to grow deeper and strengthening root vitality and nutrient absorption [5,20]. This enhanced root growth not only promotes nutrient accumulation in plant tissues but also significantly increases crop yield and quality [16,21]. In addition, micro-nano bubble irrigation may influence the symbiotic relationship between arbuscular mycorrhizal fungi (AMF) and plants. AMF form symbiotic associations with over 80% of terrestrial higher plants, significantly enhancing plants’ mineral nutrient uptake, disease resistance, and stress tolerance [11,22]. By secreting phosphatase, AMF facilitate P release from the soil, thus improving P absorption in plants and promoting growth and yield [23]. The increased oxygen level in the rhizosphere due to micro-nano bubbles may further enhance gas exchange and nutrient transfer between AMF and plant roots, supporting overall plant resilience and productivity.

Soil enzymes, especially alkaline phosphatase, play a critical role in the phosphorus metabolism between plant roots and soil microorganisms [24,25]. Alkaline phosphatase catalyzes the conversion of organic phosphorus into inorganic phosphorus, promoting efficient phosphorus uptake by plants [25,26,27]. Studies have shown that phosphatase is involved not only in the mineralization of organic phosphorus but also plays a vital role in carbon source acquisition [28]. However, under micro-nano bubble drip irrigation, the specific mechanisms by which different P levels affect maize phosphorus absorption and soil enzyme activity remain unclear. Therefore, understanding these mechanisms is crucial for optimizing P fertilizer management strategies in modern agriculture.

This study employed a field soil column cultivation method to investigate the effects of varying P application levels under micro-nano bubble water drip irrigation on maize growth, P accumulation, and soil enzyme activity. The research objectives included (1) analyzing the response of maize plant height, stem diameter, and SPAD values to phosphorus supply across different growth stages; (2) evaluating how P supply influences biomass and yield components, such as ear length, ear diameter, and grain number; (3) examining the response of soil available P, total P content, and enzyme activity to P supply under micro-nano bubble water drip irrigation; and (4) elucidating the mechanisms by which P supply enhances maize yield and P uptake under micro-nano bubble water drip irrigation. The findings of this study aim to provide a scientific basis for optimizing P fertilizer management practices in agriculture.

## 2. Materials and Methods

### 2.1. Overview of the Experimental Site

**Figure 1:**
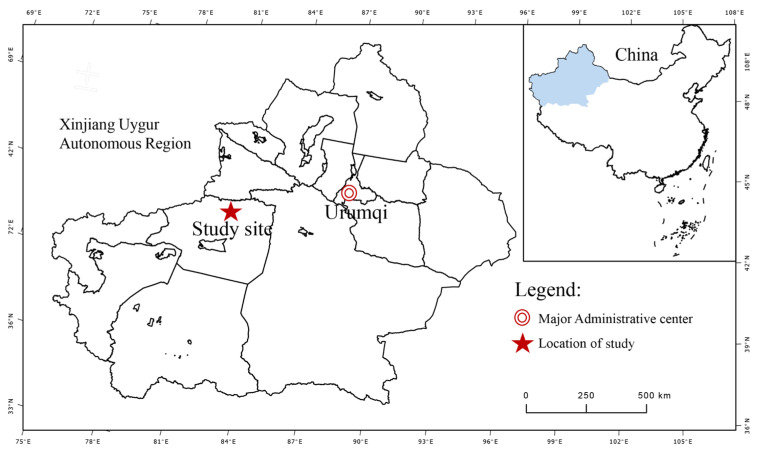
Location and layout of the study area.

The test area was situated at the base of the National Soil Quality Aksu Observation and Experiment Station in Baicheng County, Aksu Region, Xinjiang (81°54′22.6″, 41°47′37.2″, elevation 1232.1 m) (Figure 1). This region experienced a temperate continental arid climate, with an average annual temperature of 6.0 °C, an extreme max temperature of 38.3 °C, and an extreme min temperature of −28.0 °C. The frost-free period lasted between 133 and 163 d, and the mean annual sunshine duration was 2789.7 h. The average annual precipitation was 171.13 mm.

### 2.2. Field Management and Experimental Design

#### 2.2.1. Soil Collection and Preparation

The tested soil was collected from the National Soil Quality Aksu Observation and Experiment Station. The soil type was brown desert soil with a loamy sand texture, and it had not received any fertilizer for five consecutive years. Soil samples were collected from both the topsoil (0–20 cm) and subsoil (20–90 cm) layers, with the physical and chemical properties of the 0–20 cm layer shown in Table 1. The collected soil was air-dried, sieved through a 1 cm mesh, and thoroughly mixed for later use.

#### 2.2.2. Experimental Setup and Soil Column Simulation

**Figure 2:**
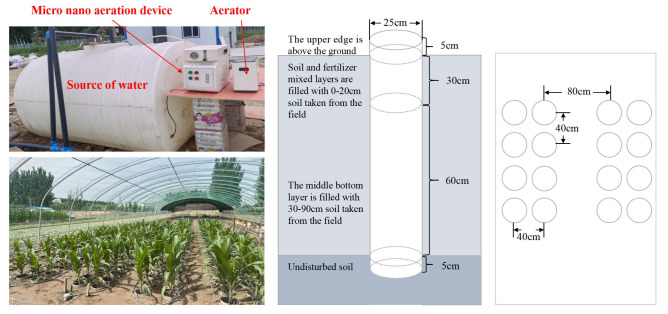
Schematic diagram of the experimental setup.

The experiment was conducted using PVC tubes with an inner diameter of 25 cm and a height of 100 cm to simulate a field cultivation environment. The top of each tube extended 5 cm above the ground to prevent surface runoff from entering after rainfall, while the bottom was left open to allow direct contact with the natural soil, simulating natural drainage conditions. Each soil column was filled with 50 kg of dry soil, divided into two layers: subsoil (30–90 cm), which was taken from the 20–90 cm field layer, and topsoil (0–30 cm), which was taken from the 0–20 cm field layer and mixed with fertilizer before being placed into the columns. After each layer was added, the soil was watered and compacted (Figure 2).

#### 2.2.3. Crop Management

The test crop was spring maize, “Tianyu 303”, which was manually sown on 1 May 2023 and harvested on 1 October 2023.

#### 2.2.4. Experimental Design

The experimental design followed a two-factor completely randomized design, with phosphorus (P) fertilizer applied at four different levels: P0 (no phosphorus fertilizer), P1 (86 kg P_2_O_5_·hm^−2^), P2 (172 kg P_2_O_5_·hm^−2^), and P3 (258 kg P_2_O_5_·hm^−2^). Additionally, two irrigation methods were used. The conventional irrigation method (I1) used surface water from the project area (dissolved oxygen = 8–9 mg·L^−1^). The micro-nano bubble oxygenated irrigation (I2) was prepared using the “B&W Micro-Nano Bubble Generator” (produced by BenZhou [Beijing, China] New Technology Promotion Co., Ltd.), operating with a working pressure of 0.015 MPa and an air intake flow rate of 1.5 L·min^−1^. To further enhance the dissolved oxygen (DO) content in the irrigation water, an oxygen supply device (YU300 model, Jiangsu Yuyue Medical Equipment Co., Ltd. Danyang, China) with an oxygen flow rate of 2 L·min^−1^ was connected to the B&W micro-nano bubble generator, providing oxygen at a concentration of 90%. Once the dissolved oxygen level stabilized, a portable dissolved oxygen meter (HQ40 model, Seven 2 Go™, Mettler Toledo International Trade Co., Ltd., Shanghai, China, ±0.1 mg·L^−1^) was used to monitor the DO concentration in the water (DO = 30 mg·L^−1^). This oxygen-enriched micro-nano bubble water was then delivered to the crop root zone via a drip irrigation system.

### 2.3. Measurement and Application Methods

#### 2.3.1. Soil Parameters

Soil samples were collected at the maize maturity stage. Six soil columns were randomly selected from each treatment, and soil samples from the 0–15 cm and 15–30 cm layers were taken using a 20-cm-diameter custom-made soil auger. After collection, the samples were quickly brought back to the laboratory, where visible fine roots, plant debris, and small stones were removed. The soil was then divided into two portions: one portion was air-dried, ground, and sieved for the determination of available phosphorus and total phosphorus; the other portion was quickly placed in an icebox for the measurement of enzyme activity. Soil total phosphorus was determined using the molybdenum antimony blue colorimetric method [29], and available phosphorus was measured using the Olsen method [30]. Alkaline protease activity was measured using an alkaline protease assay kit (BC0885, Solarbio, Suzhou, China), alkaline phosphatase activity in the soil was measured using an alkaline phosphatase assay kit (BC0285, Solarbio, Suzhou, China), and soil urease activity was measured using a urease assay kit (BC0125, Solarbio, Suzhou, China).

#### 2.3.2. Maize Plant Height, Stem Diameter, and SPAD Values

At the jointing, tasseling, filling, and maturity stages of maize, three plants were selected from each treatment. Plant height (cm) was measured using a steel tape measure, taking the height from the base of the stem to the top of the plant. Stem diameter was measured using a vernier caliper from the base of the plant at ground level. Leaf SPAD values were measured using a SPAD-502 chlorophyll meter (TOP Cloud-Agri SPAD-502, China). Measurements were taken on the uppermost fully expanded leaf at the V2 and V6 stages and on the ear leaf at the VT and R2 stages.

#### 2.3.3. Maize Biomass, Phosphorus Concentration, and Phosphorus Accumulation

At maize maturity, three plants were selected from each treatment. Biomass for different plant parts (roots, stems, leaves, cobs, husks, and grains) was determined by drying the samples at 75 °C until a constant weight was achieved. The dry weight was recorded as the biomass. The dried plant parts were then finely ground for phosphorus content analysis. Phosphorus concentration was determined using the vanadium molybdate yellow colorimetric method. The plant parts were digested with H_2_SO_4_-H_2_O_2_, and phosphorus concentration was measured spectrophotometrically at a wavelength of 440 nm [12]. Phosphorus accumulation in each organ was calculated as the phosphorus concentration multiplied by the biomass [31].

#### 2.3.4. Maize Grain Yield and Yield Components

Three maize plants from each treatment were selected for indoor yield component analysis. After natural air drying to a grain moisture content of approximately 12.5%, yield and its components were determined [32]. The number of ears per plant, the number of rows per ear, and the number of kernels per row were measured. The total weight and 100-kernel weight were recorded after threshing.

#### 2.3.5. Phosphorus Use Efficiency

The main evaluation indicators of phosphorus fertilizer use efficiency include Phosphorus Uptake Efficiency (PUE), Partial Factor Productivity of Phosphorus (PFPP), Apparent Phosphorus Recovery Efficiency (AUP), and Agronomic Phosphorus Use Efficiency (AEP), with the calculation formulas as follows [33,34,35,36]:

PUE (kg·kg^−1^) = Plant phosphorus accumulation/Applied phosphorus rate

PFPP (kg·kg^−1^) = Yield in phosphorus-applied treatment/Applied phosphorus rate

AUP (%) = [(Phosphorus accumulation in the aboveground biomass in phosphorus-applied treatment − Phosphorus accumulation in the no-phosphorus treatment) × 100]/Applied phosphorus rate

AEP (%) = [(Yield in phosphorus-applied treatment − Yield in no-phosphorus treatment) × 100]/Applied phosphorus rate

### 2.4. Statistical Analysis

The data were organized using Excel 2021, and analysis of variance (ANOVA) was conducted using SPSS Statistics 27 (IBM, Armonk, NY, USA). Two-way ANOVA was employed to assess the significant differences in various indicators under different irrigation methods and phosphorus application rates. The experimental area maps were generated using ArcGIS 10.1 (ESRI, Golden, CO, USA). Bar charts were created with Origin 2018, and structural equation modeling (SEM) analysis was performed with SPSS Amos 24 (IBM, Armonk, NY, USA) to reveal the mechanism by which oxygen-enriched irrigation and phosphorus supply contribute to maize yield improvement.

## 3. Results

### 3.1. Soil Enzyme Activity

#### 3.1.1. Alkaline Protease

**Figure 3:**
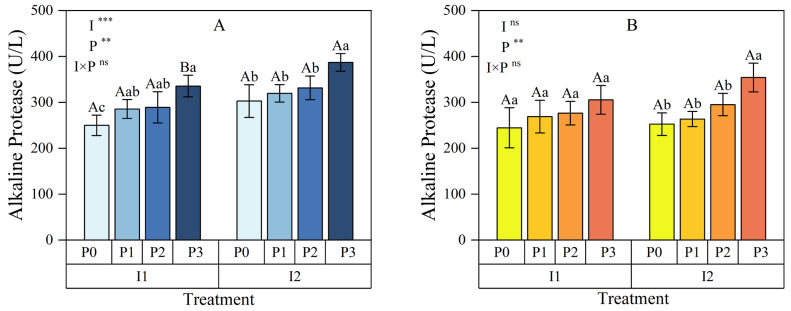
Effects of irrigation methods and phosphorus application levels on soil alkaline protease in the 0–15 cm (**A**) and 15–30 cm (**B**) soil layers. Different lowercase letters (a, b, c, …) above the bars indicate significant differences in means among different phosphorus treatments under the same irrigation method (*p* < 0.05, n = 3); different uppercase letters (A, B, C, …) indicate significant differences in means among different irrigation treatments under the same phosphorus level (*p* < 0.05, n = 3). These comparisons were conducted using one-way ANOVA and Duncan’s post hoc test. The vertical bars represent the mean ± standard deviation (SD) based on three replicates. The analysis employed two-way ANOVA, with ** and *** indicating significant differences at *p* < 0.01 and *p* < 0.001 levels, respectively, and ns indicating no significant difference. I: irrigation method; P: phosphorus application; I1: conventional irrigation; I2: micro-nano bubble water drip irrigation; P0: 0 kg·hm^−2^; P1: 86 kg·hm^−2^; P2: 172 kg·hm^−2^; P3: 258 kg·hm^−2^.

At the 0–15 cm soil depth (Figure 3A), both the irrigation method (*p* < 0.001) and phosphorus (P) application (*p* < 0.01) had a highly significant effect on soil alkaline protease activity. However, the interaction between irrigation method and P application did not significantly affect alkaline protease (ALPT) activity in this soil layer (*p* > 0.05). Within each irrigation method, soil ALPT activity increased consistently with higher P application rates. Relative to the no P application treatment (P0), ALPT activity under conventional irrigation (I1) increased by 14.20% to 34.23%, while under micro-nano bubble water drip irrigation (I2), it increased by 5.53% to 27.82%. At the same P application levels, soil ALPT activity under I2 was consistently higher than under I1, with a significant difference observed at P3 (*p* < 0.05).

In the 15–30 cm soil layer (Figure 3B), P application had a highly significant effect on soil ALPT activity (*p* < 0.01), whereas neither the irrigation method nor its interaction with P application showed significant effects (*p* > 0.05). Similarly, within each irrigation method, soil ALPT activity increased consistently with higher P application rates. Relative to P0, ALPT activity under I1 and I2 increased by 9.91% to 24.86% and 4.40% to 40.20%, respectively. Although soil ALPT activity under I2 was higher than under I1 at all P application levels, the difference was not significant.

These results suggest that I2 significantly enhanced soil ALPT activity in the 0–15 cm soil layer across all P application levels, with the greatest effect observed at higher P levels (P3). In the deeper 15–30 cm soil layer, although the effect of I2 was less pronounced, appropriate P application still effectively increased soil enzyme activity. In summary, micro-nano bubble water drip irrigation combined with appropriate P application, particularly P3, significantly boosted soil enzyme activity, improving soil health and creating favorable conditions for crop growth.

#### 3.1.2. Alkaline Phosphatase

**Figure 4:**
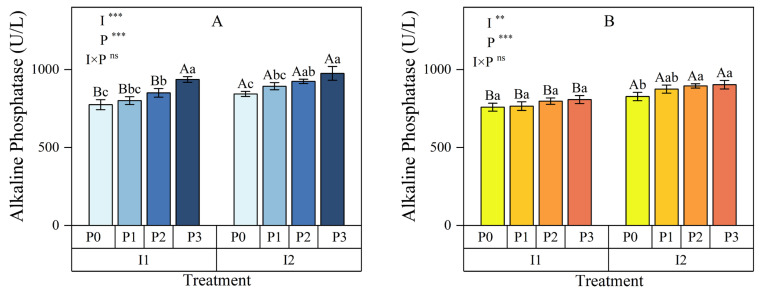
Effects of irrigation methods and phosphorus application levels on soil alkaline phosphatase in the 0–15 cm (**A**) and 15–30 cm (**B**) soil layers. Different lowercase letters (a, b, c, …) above the bars indicate significant differences in means among different phosphorus treatments under the same irrigation method (*p* < 0.05, n = 3); different uppercase letters (A, B, C, …) indicate significant differences in means among different irrigation treatments under the same phosphorus level (*p* < 0.05, n = 3). These comparisons were conducted using Duncan’s post hoc test. The vertical bars represent the mean ± standard deviation (SD) based on three replicates. The analysis employed two-way ANOVA, with ** and *** indicating significant differences at *p* < 0.01 and *p* < 0.001 levels, respectively, and ns indicating no significant difference. I: irrigation method; P: phosphorus application rate; I1: conventional irrigation; I2: micro-nano bubble water drip irrigation; P0: 0 kg·hm^−2^; P1: 86 kg·hm^−2^; P2: 172 kg·hm^−2^; P3: 258 kg·hm^−2^.

At the 0–15 cm soil depth (Figure 4A), both the irrigation method and phosphorus (P) application had highly significant effects on soil alkaline phosphatase activity (*p* < 0.01), but their interaction did not show a significant effect (*p* > 0.05). Within each irrigation method, soil alkaline phosphatase (ALP) activity consistently increased with higher P application levels. Relative to the no P application treatment (P0), ALP activity under conventional irrigation (I1) increased by 3.36% to 20.84%, while under micro-nano bubble water drip irrigation (I2), the increase ranged from 5.88% to 15.62%. At the same P application levels, soil ALP activity was 4.16% to 11.52% higher under I2 compared to I1, indicating that micro-nano bubble water drip irrigation significantly enhanced soil enzyme activity.

In the 15–30 cm soil layer (Figure 4B), the irrigation method had a highly significant effect on soil ALP activity (*p* < 0.01), and P application had an even more significant effect (*p* < 0.001); the interaction between the irrigation method and P application remained insignificant (*p* > 0.05). Similar to the surface layer, within each irrigation method, soil ALP activity progressively increased with higher P application. Compared to P0, ALP activity under I1 increased by 0.82% to 6.40%, while under I2, it increased by 5.74% to 9.16%. ALP activity under I2 was 9.00%, 14.32%, 12.26%, and 11.83% higher than under I1 for the P0, P1, P2, and P3 treatments, respectively.

These results demonstrate that I2 significantly enhanced soil ALP activity at both the 0–15 cm and 15–30 cm depths, with consistently greater increases than I1 across all P application levels. At the 0–15 cm depth, micro-nano bubble water drip irrigation combined with adequate P application (e.g., P3) significantly boosted soil ALP activity, improving P utilization and crop growth. In the 15–30 cm layer, while the effects were less pronounced than in the surface layer, appropriate micro-nano bubble water drip irrigation and P application still enhanced enzyme activity.

#### 3.1.3. Urease

**Figure 5:**
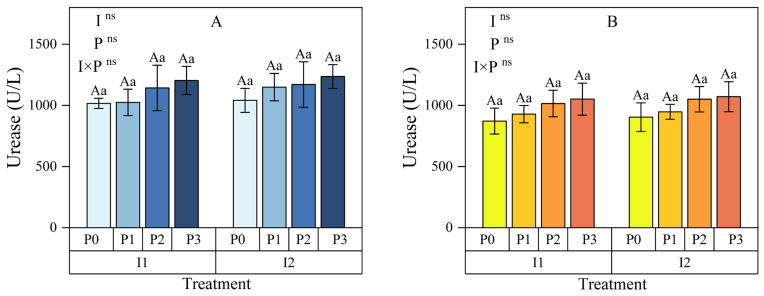
Effects of irrigation methods and phosphorus application levels on soil urease in the 0–15 cm (**A**) and 15–30 cm (**B**) soil layers. Different lowercase letters (a, b, c, …) above the bars indicate significant differences in means among different phosphorus treatments under the same irrigation method (*p* < 0.05, n = 3); different uppercase letters (A, B, C, …) indicate significant differences in means among different irrigation treatments under the same phosphorus level (*p* < 0.05, n = 3). These comparisons were conducted using Duncan’s post hoc test. The vertical bars represent the mean ± standard deviation (SD) based on three replicates. The analysis employed two-way ANOVA, with ns indicating no significant difference. I: irrigation method; P: phosphorus application rate; I1: conventional irrigation; I2: micro-nano bubble water drip irrigation; P0: 0 kg·hm^−2^; P1: 86 kg·hm^−2^; P2: 172 kg·hm^−2^; P3: 258 kg·hm^−2^.

The effects of irrigation method, phosphorus (P) application, and their interaction on soil urease (SUE) activity at the 0–15 cm depth were minimal (Figure 5A). Within each irrigation method, soil SUE activity increased with higher P application rates. Relative to no P application (P0), SUE activity under I1 increased by 0.81% to 18.46%, while under I2, the increase was 10.41% to 18.71%. At the same P levels, SUE activity under I2 was 2.43% to 12.21% higher than under I1, indicating that I2 positively impacted SUE activity.

In the 15–30 cm soil layer (Figure 5B), similarly, the effects of irrigation method, P application, and their interaction on soil SUE activity were minimal. Within each irrigation method, SUE activity consistently increased with higher P application. Relative to P0, SUE activity under I1 increased by 6.27% to 20.69%, while under I2, it increased by 4.83% to 18.46%. At the same P levels, SUE activity under I2 was 1.85% to 3.77% higher than under I1, showing that I2 also enhanced SUE activity in deeper soil, though less pronounced than in surface soil.

Although the irrigation method did not significantly influence SUE activity, I2 consistently resulted in higher activity than I1, especially at the 0–15 cm depth, where the increase was substantial. While individual factors were not significant, optimizing irrigation and P application could enhance enzyme activity, particularly in the 0–15 cm soil. Combining micro-nano bubble water drip irrigation with higher P application levels (e.g., P3) may offer a more effective management strategy.

### 3.2. Soil Available Phosphorus and Total Phosphorus

#### 3.2.1. Available Phosphorus

Irrigation method and phosphorus application significantly affected soil available phosphorus (AP) (*p* < 0.001), but their interaction was not significant (*p* > 0.05) (Table 2). At each soil depth, soil AP increased with higher phosphorus application levels. Under I1, soil AP in the 0–15 cm layer increased by 138.42% in the P3 treatment compared to P0, and by 116.67% in the 15–30 cm layer. Under I2, soil AP in the 0–15 cm layer increased by 150.58% in the P3 treatment compared to P0, and by 87.81% in the 15–30 cm layer.

At the same phosphorus level, soil AP under I2 was generally higher than under I1. In the 0–15 cm layer, AP under I2 was 9.69% to 15.58% higher than under I1, with the largest increase in the P3 treatment. In the 15–30 cm layer, AP under I2 was 1.43% to 20.72% higher than under I1, with the largest increase in the P1 treatment. This indicates that I2 significantly improved soil AP levels compared to I1, especially at higher phosphorus rates, such as P3.

#### 3.2.2. Total Phosphorus

Irrigation method and its interaction with phosphorus application had a significant effect on soil total phosphorus (TP) (*p* < 0.001), while phosphorus application alone had a weaker but significant effect (*p* < 0.05) (Table 2). Under I1, soil TP increased significantly with higher phosphorus levels. In the 0–15 cm layer, TP in the P3 treatment rose by 37.80% compared to P0, and by 39.76% in the 15–30 cm layer.

Under I2, soil TP first increased and then decreased with higher phosphorus levels. In the 0–15 cm layer, TP in the P1 treatment increased by 9.78% compared to P0, while the P3 treatment showed an 8.70% decrease compared to P0. In the 15–30 cm layer, TP in the P1 treatment rose by 3.53%, while the P3 treatment decreased by 18.82%. This suggests that increasing phosphorus application significantly boosts soil TP, particularly under conventional irrigation, where TP levels continued to rise with increasing phosphorus.

### 3.3. Maize Growth and Physiology

Irrigation method significantly affected maize height and stem diameter across growth stages (Table 3). The increase in maize height was most pronounced from the jointing to tasseling stage across irrigation methods (*p* < 0.001). Irrigation method significantly affected stem diameter at the jointing stage (*p* < 0.05), with even stronger effects at the tasseling, grain filling, and maturity stages (*p* < 0.001). SPAD values of maize under micro-nano bubble water drip irrigation (I2) were significantly higher than those under conventional irrigation (I1) at all growth stages (*p* < 0.05).

Phosphorus (P) application significantly influenced maize height and stem diameter during the jointing, tasseling, grain filling, and maturity stages (*p* < 0.01), showing an overall increasing trend, particularly between the jointing and tasseling stages. P application also significantly affected SPAD values during the jointing, tasseling, and grain filling stages (*p* < 0.01) and showed a smaller but significant effect at maturity (*p* < 0.05).

Although the interaction between irrigation method and P application did not significantly affect maize height (*p* > 0.05), it had a significant impact on stem diameter at the tasseling and grain filling stages (*p* < 0.05) and a highly significant effect at maturity (*p* < 0.001). Notably, under I2, the P3 treatment significantly increased stem diameter by 25.80%, highlighting the impact of higher P levels under micro-nano bubble water drip irrigation.

At maturity, maize height increased with higher P levels under both I1 and I2. At the same P levels, maize height under I2 was consistently higher than under I1. For instance, in the P3 treatment, maize height increased by 3.89% and 3.29% compared to P0 under I1 and I2, respectively. Under I2, maize height was 4.64%, 3.67%, 3.64%, and 4.03% higher at P0, P1, P2, and P3 levels, respectively, compared to I1.

Regarding stem diameter, P treatments (P1, P2, and P3) resulted in greater stem diameters than P0 under both I1 and I2. Under I1, stem diameter increased initially and then decreased with higher P levels, with the largest increase of 19.36% at P1 compared to P0 at maturity. In contrast, under I2, stem diameter continuously increased with higher P levels, with the largest increase of 16.49% at P3 compared to P0 at maturity. At the same P level, I2 increased stem diameter by 17.44%, 10.35%, and 25.80% compared to I1 for P0, P2, and P3, respectively, while for P1, stem diameter under I2 was 0.39% lower than under I1.

SPAD values did not differ significantly among P treatments under I1, but under I2, they increased significantly with higher P application. At the same P levels, SPAD values under I2 were higher than under I1, increasing by 2.04%, 3.65%, 11.81%, and 8.63% for P0, P1, P2, and P3 treatments, respectively.

In summary, the irrigation method, P application, and their interaction significantly affected maize height, stem diameter, and SPAD values. Micro-nano bubble water drip irrigation significantly enhanced maize growth, especially under higher P application rates, indicating its potential for improving overall crop performance.

### 3.4. Maize Biomass, Yield, and Yield Components

#### 3.4.1. Biomass

**Figure 6:**
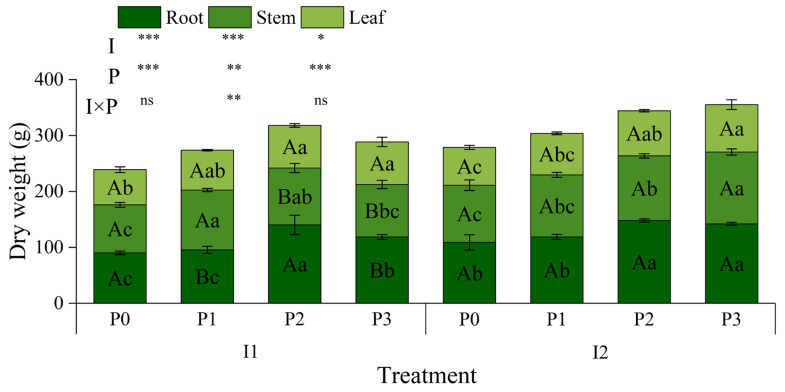
Effects of irrigation methods and phosphorus application levels on maize biomass. Different lowercase letters (a, b, c, …) indicate significant differences in means among different phosphorus treatments under the same irrigation method (*p* < 0.05, n = 3); different uppercase letters (A, B, C, …) indicate significant differences in means among different irrigation treatments under the same phosphorus condition (*p* < 0.05, n = 3). These comparisons were performed using one-way ANOVA and Duncan’s post hoc test. The vertical bars represent the mean ± standard deviation (SD) based on three replicates. The analysis employed two-way ANOVA, with *, **, and *** indicating significant differences at the *p* < 0.05, *p* < 0.01, and *p* < 0.001 levels, respectively, and ns indicating no significant difference. I: irrigation method; P: phosphorus application; I1: conventional irrigation; I2: micro-nano bubble water drip irrigation; P0: 0 kg·hm^−2^; P1: 86 kg·hm^−2^; P2: 172 kg·hm^−2^; P3: 258 kg·hm^−2^.

Irrigation method significantly affected maize root dry weight (*p* < 0.001, Figure 6), with I2 showing higher root dry weight than I1, particularly under P1 and P3 treatments. Irrigation method also significantly affected stem dry weight (*p* < 0.01) and leaf dry weight (*p* < 0.05).

Phosphorus (P) application significantly increased root, stem, and leaf dry weights (*p* < 0.01). Under the same irrigation method, P1, P2, and P3 treatments increased dry weight compared to P0, especially root dry weight, with the largest increase under P2 (55.39% for I1 and 35.99% for I2). The highest stem dry weight was observed under P1 with I1 (24.54% increase). Under I2, stem dry weight increased gradually with higher P levels, peaking at P3 (25.46% increase).

The interaction between irrigation method and P application significantly affected stem dry weight (*p* < 0.01). At the same P level, stem dry weight was higher under I2 than I1, especially under P2 and P3, suggesting that I2 more effectively promotes stem biomass accumulation.

In summary, dry weights of roots, stems, and leaves increased with higher P application under both irrigation methods, with the promoting effect of micro-nano bubble water drip irrigation (I2) being particularly pronounced.

#### 3.4.2. Yield and Yield Components

**Figure 7:**
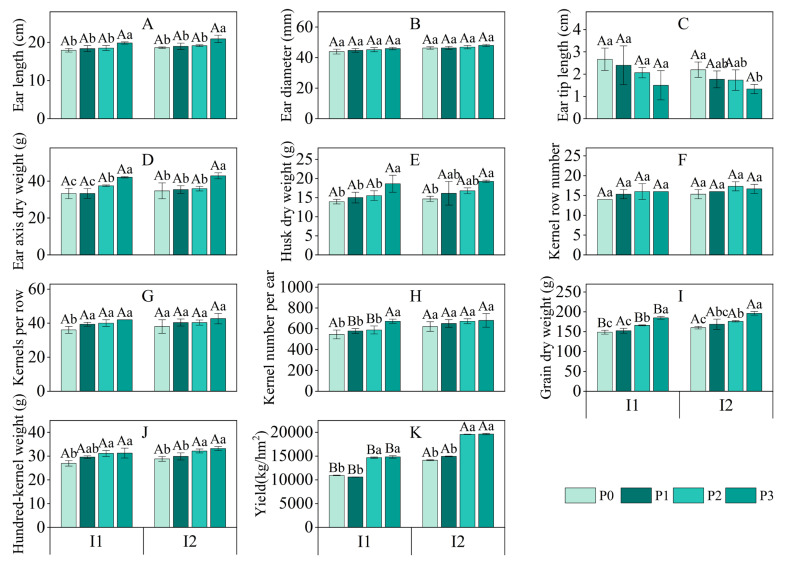
Effects of irrigation methods and phosphorus application levels on maize ear length (**A**), ear diameter (**B**), tip length (**C**), ear axis dry weight (**D**), husk dry weight (**E**), number of kernel rows (**F**), number of kernels per row (**G**), number of kernels per ear (**H**), grain dry weight (**I**), hundred-kernel weight (**J**), and yield (**K**). Different lowercase letters (a, b, c, …) indicate significant differences in means among different phosphorus treatments under the same irrigation method (*p* < 0.05, n = 3); different uppercase letters (A, B, C, …) indicate significant differences in means among different irrigation treatments under the same phosphorus condition (*p* < 0.05, n = 3). These comparisons were performed using one-way ANOVA and Duncan’s post hoc test. The vertical bars represent the mean ± standard deviation (SD) based on three replicates. I1: conventional irrigation; I2: micro-nano bubble water drip irrigation; P0: 0 kg·hm^−2^; P1: 86 kg·hm^−2^; P2: 172 kg·hm^−2^; P3: 258 kg·hm^−2^.

Irrigation method significantly affected maize grain dry weight and yield (*p* < 0.001, Table 4), with significant effects on ear length, ear diameter, and kernel number per ear (*p* < 0.01) and on kernel row number and hundred-kernel weight (*p* < 0.05). Under the same phosphorus (P) treatments, I2 resulted in increases in ear length, ear diameter, husk dry weight, kernel row number, kernels per row, kernels per ear, grain dry weight, hundred-kernel weight, and yield compared to I1. The increases for I2 compared to I1 ranged from 3.72% to 5.38% for ear length (largest increase under P3), 3.51% to 5.32% for ear diameter (largest increase under P0), 3.33% to 8.14% for husk dry weight (largest increase under P3), 4.17% to 9.52% for kernel row number (largest increase under P0), 1.59% to 5.56% for kernels per row (largest increase under P0), 1.19% to 14.29% for kernels per ear (largest increase under P3), 6.03% to 10.75% for grain dry weight (largest increase under P1), and 0.98% to 6.95% for hundred-kernel weight (largest increase under P0), with yield increasing by 29.21% to 41.08% (largest increase under P1, Figure 7).

P application significantly affected ear length, ear axis dry weight, grain dry weight, hundred-kernel weight, and yield (*p* < 0.001). It also had significant effects on husk dry weight, kernels per row, kernels per ear (*p* < 0.01), and ear tip length and kernel row number (*p* < 0.05). Under both I1 and I2, increasing P application (from P0 to P3) resulted in an upward trend in these parameters. Particularly under P3, the increases compared to P0 were 10.59% for ear length, 4.55% for ear diameter, 26.54% for ear axis dry weight, 33.56% for husk dry weight, 16.67% for kernels per row, 23.23% for kernels per ear, 24.39% for grain dry weight, and 16.01% for hundred-kernel weight under I1, and 12.37%, 3.76%, 23.44%, 31.35%, 12.28%, 9.56%, 22.54%, and 15.15% under I2, respectively.

The interaction between irrigation method and P application significantly affected yield (*p* < 0.001). Under I1, the P1 treatment slightly reduced yield compared to P0 (by 3.16%), while P2 and P3 treatments significantly increased yield (by 34.14% and 35.66%, respectively). Under I2, yield showed a significant increasing trend with higher P application levels (with increases of 5.73%, 38.41%, and 38.88% for P1, P2, and P3, respectively, compared to P0).

In summary, increasing P application positively influenced maize grain dry weight and kernel number per ear, while micro-nano bubble water drip irrigation further enhanced these effects, particularly in yield.

### 3.5. Maize Phosphorus Concentration and Phosphorus Accumulation

#### 3.5.1. Phosphorus Concentration

**Figure 8:**
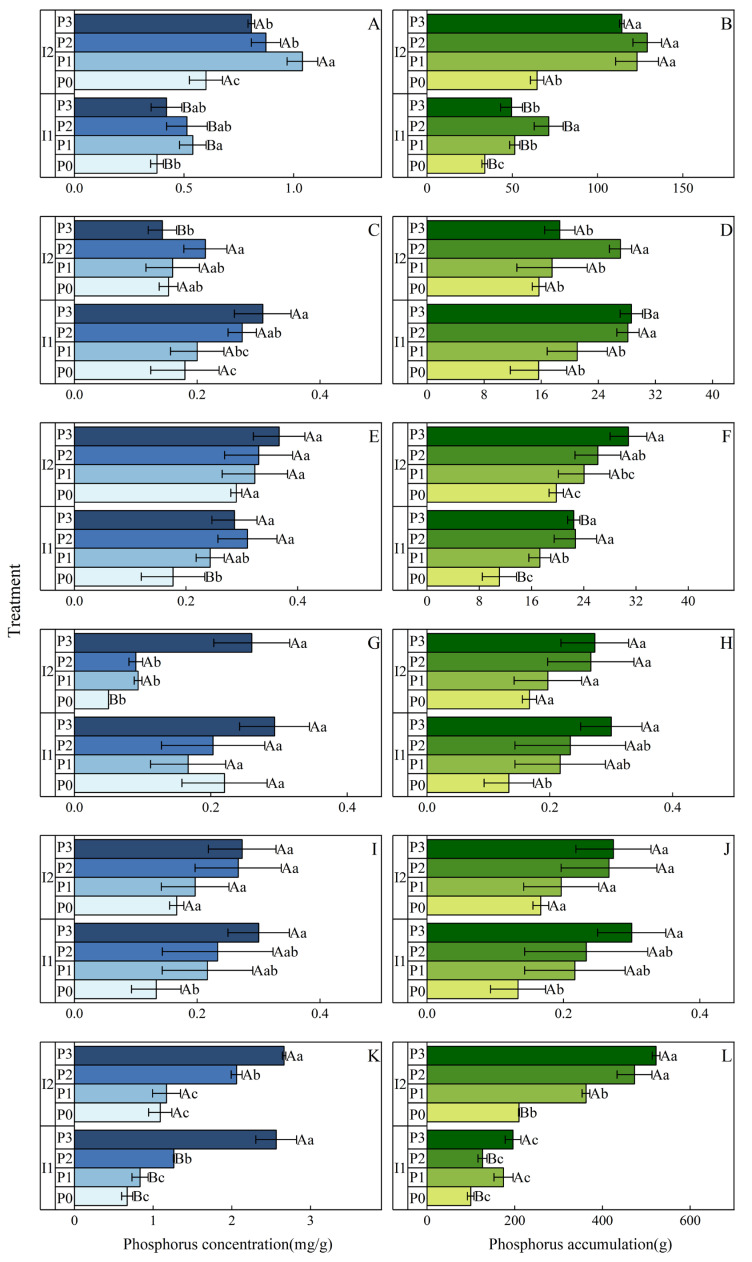
Effects of irrigation methods and phosphorus application levels on phosphorus concentration in maize roots (**A**), phosphorus accumulation in roots (**B**), phosphorus concentration in stems (**C**), phosphorus accumulation in stems (**D**), phosphorus concentration in leaves (**E**), phosphorus accumulation in leaves (**F**), phosphorus concentration in husks (**G**), phosphorus accumulation in husks (**H**), phosphorus concentration in ear axes (**I**), phosphorus accumulation in ear axes (**J**), phosphorus concentration in grains (**K**), and phosphorus accumulation in grains (**L**). In the bar graph, different lowercase letters (a, b, c, …) indicate significant differences in the mean values of different phosphorus treatments under the same irrigation method (*p* < 0.05, n = 3); different uppercase letters (A, B, C, …) indicate significant differences in the mean values of different irrigation treatments under the same phosphorus application (*p* < 0.05, n = 3). These comparisons were conducted using one-way ANOVA and Duncan’s post hoc test. The bars represent the mean values ± standard deviation (SD) based on three replicates. I1: conventional irrigation; I2: micro-nano bubble water drip irrigation; P0: 0 kg·hm^−2^; P1: 86 kg·hm^−2^; P2: 172 kg·hm^−2^; P3: 258 kg·hm^−2^.

Irrigation method significantly affected phosphorus (P) concentrations in roots, leaves, husks, and grains (*p* < 0.001, Table 5) and had a significant effect on stems (*p* < 0.01) but no significant effect on ear axes (*p* > 0.05). At the same P application levels, I2 treatment resulted in higher P concentrations in roots and grains compared to I1, with increases of 59.28% to 92.59% (largest under P1) and 3.90% to 63.06% (largest under P3), respectively. Conversely, P concentrations in stems and husks under I2 were significantly lower than under I1, with decreases of 14.83% to 53.28% (largest under P3) and 11.35% to 77.27% (largest under P0), respectively (Figure 8).

P application levels had a highly significant effect on P concentrations in roots, stems, leaves, ear axes, husks, and grains (*p* < 0.001). Under the same irrigation method, as P application increased, the P concentrations in roots, leaves, and grains showed an increasing trend. Increasing P application significantly raised P concentrations in ear axes and grains. Under I1, the P3 treatment resulted in increases of 125.06% for ear axis and 282.58% for grain P concentrations; under I2, the increases were 63.95% and 144.34%, respectively.

The interaction between irrigation method and P application significantly affected P concentration in roots (*p* < 0.001) and had a significant effect on stems (*p* < 0.05). In both I1 and I2, P concentration in roots increased initially and then decreased with higher P application, peaking at P1. The P concentration in leaves exhibited an initial increase followed by a decrease under I1, while under I2, it continued to increase with rising P application, with a 26.45% increase under P3 compared to P0. This trend might suggest that the mechanisms of P uptake and distribution in plants could differ under different irrigation methods.

#### 3.5.2. Phosphorus Accumulation

Irrigation method significantly affected phosphorus (P) accumulation in roots, stems, husks, and grains (*p* < 0.001, Table 5) and had a significant effect on P accumulation in leaves (*p* < 0.01) but no significant effect on ear axes (*p* > 0.05). At the same P application levels, P accumulation in roots under I2 was significantly higher than under I1, with increases ranging from 81.07% to 139.53% (largest under P1). In contrast, P accumulation in stems was higher under I2 only at P0 (an increase of 0.39%), while at other P levels, it was lower than under I1 (Figure 8).

P application levels had a highly significant effect on P accumulation in roots, husks, and grains (*p* < 0.001) and a significant effect on P accumulation in stems and ear axes (*p* < 0.01) while having a significant effect on P accumulation in leaves (*p* < 0.05). Under the same irrigation method, as P application increased, overall P accumulation in roots, stems, leaves, ear axes, and grains showed an increasing trend. Under I1, P accumulation in ear axes for the P3 treatment increased by 185.88% compared to P0, while under I2, the increase for the same treatment was 101.30%.

The interaction between irrigation method and P application significantly affected P accumulation in grains (*p* < 0.01) and had a significant effect on P accumulation in roots and stems (*p* < 0.05). In both I1 and I2, P accumulation in grains increased with increasing P application. For the P3 treatment, P accumulation in grains under I1 increased by 96.82% compared to P0, while under I2, the increase was 149.15%.

### 3.6. Utilization Efficiency of Phosphorus in Maize

Irrigation methods significantly influenced phosphorus uptake efficiency (PUE) and agronomic efficiency of phosphorus fertilizer (AEP) (*p* < 0.001), as well as partial factor productivity of phosphorus fertilizer (PFPP) (*p* < 0.01) (Table 6). Phosphorus application rates also significantly affected PUE and AEP (*p* < 0.001), with notable effects on PFPP and the apparent utilization rate of phosphorus fertilizer (AUP) (*p* < 0.01). Additionally, there was a highly significant interaction between irrigation method and phosphorus application rate on AEP (*p* < 0.001) and a significant effect on phosphorus absorption efficiency (*p* < 0.01).

Under conventional irrigation (I1), PUE values for the P1, P2, and P3 treatments were 0.54 kg·kg^−1^, 0.41 kg·kg^−1^, and 0.47 kg·kg^−1^, respectively. Under micro-nano bubble water drip irrigation (I2), PUE demonstrated a decreasing trend with increasing phosphorus application rates, with values of 0.88 kg·kg^−1^ for P1, 0.65 kg·kg^−1^ for P2, and 0.52 kg·kg^−1^ for P3. At the same phosphorus application levels, PUE was higher under I2 compared to I1, with increases of 62.96% for P1, 58.54% for P2, and 10.64% for P3. The differences were statistically significant for P1 and P2 (*p* < 0.05).

PFPP initially increased and then decreased with increasing phosphorus application under both irrigation methods, reaching a maximum at the P2 level. The PFPP for P2 was 2.46 kg·kg^−1^ under I1 and 3.07 kg·kg^−1^ under I2. PFPP under I2 was significantly higher than under I1, with increases of 22.99%, 24.80%, and 84.33% for P1, P2, and P3, respectively (*p* < 0.05).

AEP also followed a pattern of initial increase followed by a decrease with increasing phosphorus application, reaching its peak at the P2 level. AEP for P2 was 21.71% under I1 and 31.57% under I2. AEP under I2 was significantly higher than under I1, with increases of 134.91%, 45.42%, and 40.87% for P1, P2, and P3, respectively (*p* < 0.05).

AUP showed an increasing trend with rising phosphorus application under both irrigation methods. For the P3 treatment, AUP values were 32.26% under I1 and 28.78% under I2. AUP under I2 was significantly higher than under I1 at the P2 application level, with an increase of 50.27% (*p* < 0.05). However, AUP values for the P1 and P3 levels were lower under I2 compared to I1, with reductions of 26.67% and 10.79%, respectively.

In summary, micro-nano bubble water drip irrigation significantly enhanced phosphorus uptake efficiency, partial factor productivity, and agronomic efficiency of phosphorus fertilizer, particularly at the P1 and P2 application levels. Although the impact on the apparent utilization rate of phosphorus fertilizer was generally modest, there was a notable increase under the P2 treatment. This suggests that micro-nano bubble water drip irrigation could effectively improve phosphorus utilization efficiency, particularly at moderate phosphorus application rates.

### 3.7. Structural Equation Model

**Figure 9:**
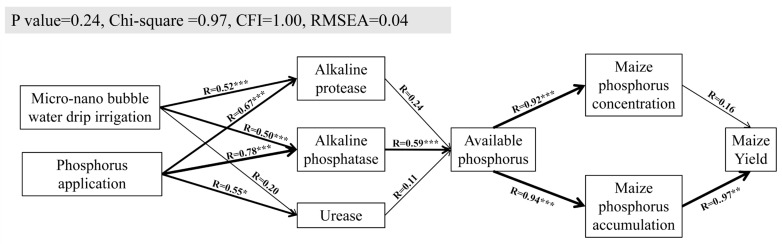
Structural equation model of micro-nano bubble water drip irrigation and phosphorus application levels on maize phosphorus uptake and yield enhancement. The thickness of the lines represents the strength of the correlation. *, **, and *** indicate significance levels at *p* < 0.05, *p* < 0.01, and *p* < 0.001, respectively.

A structural equation model (SEM) was used to analyze how micro-nano bubble water drip irrigation promotes maize yield through phosphorus supply (Figure 9) (*p*-value = 0.24, Chi-square = 0.97, CFI = 1.00, RMSEA = 0.04). The results showed that alkaline protease and alkaline phosphatase had the strongest responses to micro-nano bubble water drip irrigation, while all three enzymes—alkaline protease, alkaline phosphatase, and urease—responded well to phosphorus application. The standardized path coefficients for alkaline protease and alkaline phosphatase were notably higher, indicating their greater sensitivity to phosphorus supply. Additionally, available phosphorus exhibited a significant response to alkaline phosphatase, and maize phosphorus concentration and accumulation responded strongly to available phosphorus. Furthermore, phosphorus accumulation significantly promoted maize yield.

The model confirmed that, under micro-nano bubble water drip irrigation, phosphorus supply primarily regulates alkaline protease and alkaline phosphatase, enhancing soil phosphorus availability. This, in turn, promotes phosphorus absorption and accumulation, ultimately increasing maize yield. This mechanism supports the integration of micro-nano bubble water drip irrigation with phosphorus management to enhance crop productivity.

## 4. Discussion

### 4.1. Soil Enzyme Activity and Phosphorus Accumulation

The movement of phosphorus (P) in the soil primarily depends on convection and adsorption mechanisms, with its availability significantly influenced by soil microorganisms and enzyme activity [9,13,37,38]. Our study showed that irrigation methods and P application rates significantly impact soil enzyme activity, which not only affects P transformation efficiency but is also closely related to the carbon and nitrogen cycles in the soil. For instance, we observed that micro-nano bubble water drip irrigation significantly increased alkaline phosphatase and alkaline protease activities in the 0–15 cm soil layer. This indicates that micro-nano bubble water drip irrigation improves the soil microbial environment, promoting the proliferation of aerobic microorganisms [5,39,40]. These aerobic microorganisms accelerate the decomposition of organic matter and the release of nutrients, facilitating the conversion of organic P to inorganic P, thereby enhancing P availability in the soil [16,41]. This process effectively improves the dynamic balance of P in the soil, enabling crops to absorb P more efficiently. Our findings align with previous studies, further demonstrating that micro-nano bubble water drip irrigation can enhance P use efficiency, particularly under conditions of limited water resources and low P input [13].

Additionally, our study revealed that different P application strategies significantly influence the movement and availability of P in the soil. Excessive P application may lead to the accumulation and even fixation of P, reducing its utilization efficiency [7,28,37,38]. However, moderate P application combined with micro-nano bubble water drip irrigation prevents excessive P buildup in the soil, maintains higher P availability, and ensures that plants can efficiently absorb and utilize P [17]. This finding is consistent with our results, further proving that a reasonable combination of P application rates and irrigation methods can effectively improve soil P utilization efficiency, thereby optimizing crop production.

### 4.2. Maize Growth and Biomass Distribution in Response to Irrigation and Phosphorus Application

Different irrigation methods significantly impacted maize growth, as evidenced by growth parameters such as plant height, stem diameter, and leaf SPAD values. Specifically, the plant height of maize under micro-nano bubble water drip irrigation (I2) treatment (233–240.67 cm) was notably higher than that under conventional irrigation (I1) treatment (222.67–231.33 cm). Similarly, both stem diameter (I2: 31.69–36.92 mm vs. I1: 26.98–32.21 mm) and SPAD values (I2: 45.1–48.27 vs. I1: 44.2–44.43) were enhanced in I2 compared to I1. These results indicate that micro-nano bubble water drip irrigation not only improved the root growth environment of maize but also enhanced photosynthetic efficiency, thereby boosting overall crop performance. This finding aligns with previous research, which suggests that micro-nano bubble water drip irrigation effectively promotes plant growth by improving soil aeration and phosphorus (P) availability [17,39,42,43]. The improved oxygen supply in the soil enhances root vitality, leading to better absorption of water and nutrients and subsequently increasing overall biomass [5,13,40]. We observed that micro-nano bubble water drip irrigation significantly increased the dry weight of maize organs, which can be attributed to improved root growth and higher nutrient absorption efficiency [16,17].

Additionally, the increase in P application had a significant effect on maize growth parameters [1,9]. The study found that appropriate P application markedly enhances photosynthetic efficiency and plant vigor [44]. For instance, under conventional irrigation, maize stem diameter in the high P treatment (P3: 29.35 mm) was greater than that in both the low P (P1: 32.21 mm) and medium P (P2: 30.25 mm) treatments. Under micro-nano bubble water drip irrigation (I2), maize stem diameter increased with higher P application, indicating a synergistic effect between micro-nano bubble water drip irrigation and P fertilizer in promoting maize growth [17,43].

### 4.3. Impact of Irrigation Methods and Phosphorus Application on Maize Yield and Components

Irrigation methods and phosphorus (P) application not only influenced the biomass distribution of maize but also significantly affected its yield and yield components. This study demonstrated that, under the same P application levels, the micro-nano bubble water drip irrigation (I2) treatment resulted in a significant yield increase (by 29.21%–41.08%) compared to conventional irrigation (I1), indicating a higher yield potential. Additionally, at the same P application level, both the P concentration and accumulation in the roots and grains of maize under micro-nano bubble water drip irrigation were significantly higher than those observed under conventional irrigation. This suggests that micro-nano bubble water drip irrigation improved oxygen supply to the roots, thereby enhancing P absorption and transport efficiency, which ultimately increased P content in maize organs [17]. These findings align with previous studies, highlighting the significant advantages of micro-nano bubble water drip irrigation for P uptake and utilization.

Moreover, the increase in P application had a notable effect on maize yield, grain P concentration, and overall P content [44]. In the P3 (high P) treatment, maize yield, grain P concentration, and P content were significantly higher than those in the P1 (low P) treatment. This indicates that as P application increased, P supply in the soil became more adequate, allowing maize to absorb P more effectively, thereby enhancing P accumulation and yield in the grains [45,46].

Furthermore, micro-nano bubble water drip irrigation exhibited selectivity in P distribution. The results suggest that this irrigation method may preferentially promote the transfer of P to the grains while reducing its accumulation in the stems and husks. This preferential allocation mechanism contributed to increased P content in the grains, ultimately improving crop yield [8]. In contrast, under conventional irrigation, higher P content was observed in the stems and husks of maize. This finding indicates that micro-nano bubble water drip irrigation not only influenced P distribution across maize organs but also optimized P allocation efficiency within the crop.

### 4.4. Enhancing Phosphorus Uptake and Utilization Efficiency through Irrigation Methods

Phosphorus uptake efficiency (PUE) and agronomic phosphorus use efficiency (AEP) are critical indicators that determine maize yield and resource utilization [13,28,41]. This study found that micro-nano bubble water drip irrigation (I2) significantly improved maize PUE and AEP, particularly under low phosphorus (P1) and medium phosphorus (P2) treatments. Specifically, under different phosphorus application levels, the PUE, phosphorus partial productivity, and AEP in micro-nano bubble water drip irrigation were consistently higher than those observed with conventional irrigation (I1). This indicates that micro-nano bubble water drip irrigation enhances yield and promotes sustainable agriculture by improving phosphorus fertilizer utilization efficiency, especially when resources are limited.

Under low phosphorus conditions, the AEP of micro-nano bubble water drip irrigation was significantly greater than that of conventional irrigation. For instance, in the P1 treatment, the AEP of I2 was 9.42%, significantly higher than the 4.01% observed with I1. Similarly, at medium phosphorus levels, the AEP of I2 reached 31.57%, which was substantially higher than the 21.71% recorded for I1. These results demonstrate that micro-nano bubble water drip irrigation can effectively enhance maize yield without necessitating increased phosphorus fertilizer input.

Previous studies have indicated that micro-nano bubble water drip irrigation improves oxygen supply to the roots, thereby enhancing root activity and increasing phosphorus uptake capacity [17]. Additionally, micro-nano bubble water drip irrigation significantly increased the apparent phosphorus use efficiency. In the P2 treatment, the apparent phosphorus use efficiency of I2 was 24.66%, markedly higher than the 16.41% of I1. However, under high phosphorus (P3) levels, the difference in apparent phosphorus use efficiency between I2 (28.78%) and I1 (32.26%) was not significant. This likely occurred because, under high phosphorus conditions, the soil already had an adequate phosphorus supply, which diminished the enhancing effect of micro-nano bubble water drip irrigation on phosphorus fertilizer utilization efficiency.

In summary, despite variations in apparent phosphorus use efficiency among treatments, micro-nano bubble water drip irrigation significantly improved phosphorus uptake and fertilizer utilization efficiency, particularly under low and medium phosphorus levels. This suggests that, in scenarios with limited water and phosphorus resources, implementing a reasonable irrigation strategy—especially micro-nano bubble water drip irrigation—can substantially increase maize yield and enhance the efficient use of limited resources.

### 4.5. Mechanisms of Phosphorus Supply in Enhancing Maize Yield with Micro-Nano Bubble Water Drip Irrigation

Micro-nano bubble water drip irrigation increases soil oxygen content, which promotes aerobic respiration in plant roots and enhances soil enzyme activity. These changes ultimately lead to a significant increase in crop yield [17,20,39,40,42]. This positive effect has been validated in maize [47], rice [48], and vegetables [17]. However, the mechanisms underlying the interaction between phosphorus (P) and oxygen in improving crop yield need further refinement. In this study, structural equation modeling (SEM) revealed that, under micro-nano bubble water drip irrigation, P supply primarily regulates alkaline protease and alkaline phosphatase. This regulation increases soil P availability, which in turn promotes P accumulation in maize and enhances yield. These findings align with previous studies, underscoring the significant benefits of P-oxygen interactions for improving both yield and P uptake [49,50].

Currently, research on oxygen deficiency in the root zone, its effect on crop yield enhancement, and the mechanisms promoting P absorption is limited. By investigating the “P-oxygen interaction” in maize, this study provides a theoretical foundation for enhancing crop yield and P use efficiency while reducing the need for P fertilizer application.

## 5. Conclusions

Micro-nano bubble water drip irrigation significantly enhanced maize growth parameters, including plant height, stem diameter, leaf SPAD values, and biomass, by improving soil oxygen supply. This enhancement boosted phosphorus uptake capacity, which in turn promoted growth and photosynthetic efficiency. Furthermore, this irrigation method markedly increased maize yield, phosphorus uptake efficiency, partial factor productivity of phosphorus, and agronomic phosphorus use efficiency. These benefits were especially pronounced under low and medium phosphorus levels, where the method facilitated phosphorus accumulation and optimized nutrient distribution in the grains, ultimately leading to higher yields. Additionally, moderate increases in phosphorus application further enhanced grain phosphorus concentration and content, thereby improving the crop’s phosphorus utilization efficiency. Structural equation modeling indicated that phosphorus supply primarily regulated alkaline protease and alkaline phosphatase activities under micro-nano bubble water drip irrigation, resulting in increased soil phosphorus availability and enhanced phosphorus accumulation in maize. This combined strategy offers an effective approach for boosting crop yields, optimizing water and fertilizer resource use, and minimizing phosphorus waste and environmental pollution. Therefore, it is recommended that agricultural production, particularly in resource-limited environments, adopt the management strategy of micro-nano bubble water drip irrigation combined with moderate phosphorus application to achieve high efficiency and sustainable agricultural goals.

## Figures and Tables

**Table 1:** Physicochemical properties of rhizosphere soil.

PH	Water-Soluble Salt (g·kg^−1^)	Hydrolyzable Nitrogen (mg·kg^−1^)	Available Phosphorus (mg·kg^−1^)	Potassium (mg·kg^−1^)	Organic Matter (g·kg^−1^)	Total Nitrogen (g·kg^−1^)	Carbon-Nitrogen Ratio (%)
8.12	3.1	107	14.7	409	27.2	1.68	16.17

**Table 2:** Effect of irrigation methods and phosphorus application levels on soil available phosphorus and total phosphorus.

Index	Irrigation Method	Phosphorus Application Level	Soil Layer (cm)	
			0–15	15–30
Available Phosphorus (mg·kg^−1^)	I1	P0	6.22 ± 0.64 Ac	5.82 ± 1.33 Ad
		P1	9.7 ± 2.15 Ab	8.11 ± 0.77 Bc
		P2	11.51 ± 0.7 Bb	10.18 ± 0.24 Ab
		P3	14.83 ± 1.5 Aa	12.61 ± 0.71 Aa
	I2	P0	6.84 ± 0.43 Ad	6.81 ± 0.34 Ac
		P1	10.64 ± 0.35 Ac	9.79 ± 0.68 Ab
		P2	13.14 ± 0.1 Ab	10.49 ± 1 Ab
		P3	17.14 ± 0.31 Aa	12.79 ± 0.73 Aa
	I		16.91 ***	
	P		159.42 ***	
	I × P		0.2 ^ns^	
Total Phosphorus (mg·kg^−1^)	I1	P0	0.82 ± 0.06 Ac	0.83 ± 0.11 Ab
		P1	0.91 ± 0.17 Abc	0.86 ± 0.06 Ab
		P2	1.03 ± 0.05 Bab	0.96 ± 0.1 Aab
		P3	1.13 ± 0.06 Aa	1.16 ± 0.1 Ba
	I2	P0	0.92 ± 0.09 Aab	0.85 ± 0.09 Aab
		P1	1.01 ± 0.02 Aa	0.88 ± 0.11 Aa
		P2	0.91 ± 0.03 Ab	0.86 ± 0.03 Aab
		P3	0.84 ± 0.02 Bb	0.69 ± 0.06 Ab
	I		14.86 ***	
	P		3.52 *	
	I × P		18.56 ***	

Note: Different lowercase letters (a, b, c, …) indicate significant differences in means among different phosphorus treatments under the same irrigation method (*p* < 0.05, n = 3); different uppercase letters (A, B, C, …) indicate significant differences in means among different irrigation treatments under the same phosphorus level (*p* < 0.05, n = 3), assessed using one-way ANOVA and Duncan’s post hoc test. Data are presented as mean ± standard deviation (SD) based on three replicates. The analysis employed two-way ANOVA, with * and *** indicating significant differences at the *p* < 0.05 and *p* < 0.001 levels, respectively, and ^ns^ indicating no significant difference. I: irrigation method; P: phosphorus application; I1: conventional irrigation; I2: micro-nano bubble water drip irrigation; P0: 0 kg·hm^−2^; P1: 86 kg·hm^−2^; P2: 172 kg·hm^−2^; P3: 258 kg·hm^−2^.

**Table 3:** Effects of irrigation methods and phosphorus application levels on maize plant height, stem diameter, and SPAD.

Index	Irrigation Method	Phosphorus Application	Jointing Stage	Tasseling Stage	Filling Stage	Maturity Stage
Plant Height (cm)	I1	P0	51.67 ± 1.15 Bb	209.33 ± 6.51 Ab	215 ± 4 Bc	222.67 ± 2.08 Bb
		Pl	53.33 ± 1.53 Bb	215 ± 4.36 Aab	220.33 ± 5.03 Bbc	227.33 ± 2.89 Ba
		P2	59 ± 2 Ba	217.33 ± 4.04 Bab	223.67 ± 3.06 Bab	229 ± 1.73 Ba
		P3	60.67 ± 2.31 Ba	220.67 ± 2.89 Ba	229.33 ± 1.53 Ba	231.33 ± 1.53 Ba
	I2	P0	60.33 ± 3.21 Ac	214 ± 4.58 Ab	228.33 ± 2.52 Ab	233 ± 1.73 Ac
		P1	63 ± 1 Abc	217 ± 4 Ab	230.33 ± 3.21 Ab	235.67 ± 1.53 Abc
		P2	66.67 ± 2.52 Aab	224 ± 1 Aa	231 ± 2.65 Ab	237.33 ± 2.52 Aab
		P3	70.33 ± 3.21 Aa	225.67 ± 1.15 Aa	239.33 ± 1.53 Aa	240.67 ± 2.08 Aa
	I		93.08 ***	8.05 *	62.08 ***	116.48 ***
	P		21.88 ***	9.94 **	17.22 ***	16.43 ***
	I × P		0.27 ^ns^	0.36 ^ns^	0.92 ^ns^	0.32 ^ns^
Stem Diameter (mm)	I1	P0	16.98 ± 0.91 Bb	23.98 ± 0.91 Bb	24.65 ± 1.43 Bb	26.98 ± 0.91 Bc
		P1	23.08 ± 2.06 Aa	27.07 ± 1.14 Aa	28.42 ± 0.02 Ba	32.21 ± 0.69 Aa
		P2	21.7 ± 3.34 Aa	26.77 ± 1.05 Aa	28.7 ± 0.72 Ba	30.25 ± 0.7 Bb
		P3	19.74 ± 1.43 Bab	25.41 ± 0.58 Bab	27.07 ± 1.14 Ba	29.35 ± 1.09 Bb
	I2	P0	20.45 ± 1.42 Ab	27.06 ± 0.03 Ab	29.02 ± 0.28 Ac	31.69 ± 1.39 Ac
		P1	21.64 ± 0.82 Aab	27.99 ± 0.02 Ab	29.51 ± 0.53 Abc	32.08 ± 0.14 Abc
		P2	22.13 ± 0.27 Aab	28.23 ± 0.21 Aab	30.85 ± 1.12 Aab	33.38 ± 0.46 Ab
		P3	23.38 ± 1.42 Aa	29.72 ± 1.45 Aa	31.35 ± 1.07 Aa	36.92 ± 0.76 Aa
	I		4.82 *	49.99 ***	64.29 ***	122.20 ***
	P		5.64 **	8.46 **	12.07 ***	21.81 ***
	I×P		3.17 ^ns^	4.99 *	4.76 *	21.56 ***
SPAD	I1	P0	39.87 ± 1.21 Bb	44.87 ± 1.21 Bb	49.87 ± 1.21 Ba	44.2 ± 2.31 Aa
		P1	41.13 ± 0.06 Bab	46.13 ± 0.06 Bab	50.8 ± 0.61 Ba	43.8 ± 0.61 Aa
		P2	41.4 ± 0.75 Bab	46.4 ± 0.75 Bab	51.07 ± 1 Ba	43.73 ± 1.45 Ba
		P3	42.1 ± 1.01 Ba	47.1 ± 1.01 Ba	51.1 ± 2.15 Ba	44.43 ± 1.08 Ba
	I2	P0	42.07 ± 0.4 Ab	47.73 ± 0.51 Ab	52.73 ± 0.51 Ab	45.1 ± 1.21 Ab
		P1	43.1 ± 1.21 Ab	48.1 ± 1.21 Ab	53.1 ± 1.21 Ab	45.4 ± 1.01 Ab
		P2	46.23 ± 0.99 Aa	51.23 ± 0.99 Aa	56.23 ± 0.99 Aa	48.9 ± 0.8 Aa
		P3	47.27 ± 0.55 Aa	51.93 ± 0.78 Aa	57.27 ± 2.12 Aa	48.27 ± 0.84 Aa
	I		100.91 ***	99.07 ***	55.69 ***	31.00 ***
	P		22.45 ***	16.64 ***	6.12 **	3.65 *
	I × P		5.76 **	3.92 *	2.77 ^ns^	3.66 *

Note: Different lowercase letters (a, b, c, …) indicate significant differences in means among different phosphorus treatments under the same irrigation method (*p* < 0.05, n = 3); different uppercase letters (A, B, C, …) indicate significant differences in means among different irrigation treatments under the same phosphorus level (*p* < 0.05, n = 3), assessed using one-way ANOVA and Duncan’s post hoc test. Data are presented as mean ± standard deviation (SD) based on three replicates. The analysis employed two-way ANOVA, with *, **, and *** indicating significant differences at the *p* < 0.05, *p* < 0.01 and *p* < 0.001 levels, respectively, and ^ns^ indicating no significant difference. I: irrigation method; P: phosphorus application; I1: conventional irrigation; I2: micro-nano bubble water drip irrigation; P0: 0 kg·hm^−2^; P1: 86 kg·hm^−2^; P2: 172 kg·hm^−2^; P3: 258 kg·hm^−2^.

**Table 4:** Variance analysis of maize yield index by irrigation methods and phosphorus application levels.

Variance	Ear Length	Ear Diameter	Ear Tip Length	Ear Axis Dry Weight	Husk Dry Weight	Kernel Row Number	Kernel Per Row	Kernel Number Per Ear	Grain Dry Weight	Hundred-Kernel Weight	Yield
I	8.55 **	17.59 **	3.81 ^ns^	0.55 ^ns^	2.08 ^ns^	5.14 *	1.16 ^ns^	13.64 **	26.42 ***	6.24 *	6674.30 ***
P	13.2 ***	3.05 ^ns^	4.27*	17.33 ***	9.44 **	4 *	5.57 **	5.71 **	43.89 ***	15.18 ***	2458.98 ***
I × P	0.19 ^ns^	0.15 ^ns^	0.23 ^ns^	0.72 ^ns^	0.06 ^ns^	0.19 ^ns^	0.15 ^ns^	1.16 ^ns^	0.34 ^ns^	0.6 ^ns^	54.32 ***

Note: This table used two-way analysis of variance (ANOVA) to evaluate the significant differences between treatments. The data in the table represent F values, with *, **, and *** indicating significant differences at *p* < 0.05, *p* < 0.01, and *p* < 0.001, respectively, while ^ns^ indicates no significant difference. I: irrigation method; P: phosphorus application.

**Table 5:** Variance analysis of phosphorus concentration and phosphorus accumulation in maize by irrigation methods and phosphorus application levels.

Index	Phosphorus Concentration			Phosphorus Accumulation		
	I	P	I × P	I	P	I × P
Root	131.67 **	15.89 **	3.15 *	408.88 ***	61.02 ***	10.42 ***
Stem	23.08 **	5.68 **	4.01 *	9.01 **	18.40 ***	3.48 *
Leaf	14.61 **	4.98 *	1.03 ^ns^	38.94 ***	19.94 ***	1.20 ^ns^
Bract	0.04 ^ns^	5.76 **	0.45 ^ns^	0.142 ^ns^	14.08 ***	0.42 ^ns^
Cob	24.62 **	12.72 **	2.2 ^ns^	26.69 ***	40.15 ***	2.66 ^ns^
Grain	15.48 **	57.13 **	1.9 ^ns^	132.28 ***	484.52 ***	9.21 **

Note: This table used two-way analysis of variance (ANOVA) to evaluate the significant differences between treatments. The data in the table represent F values, with *, **, and *** indicating significant differences at *p* < 0.05, *p* < 0.01, and *p* < 0.001, respectively, while ^ns^ indicates no significant difference. I: irrigation method; P: phosphorus application.

**Table 6:** Phosphorus utilization efficiency of maize under different irrigation methods and phosphorus application levels.

Irrigation Method	Phosphorus Application Rate	PUE (kg·kg^−1^)	PFPP (kg·kg^−1^)	AUP (%)	AEP (%)
I1	P0	/	/	/	/
	P1	0.54 ± 0.01 Ba	1.87 ± 0.03 Bc	9.6 ± 3.19 Ac	4.01 ± 0.97 Bc
	P2	0.41 ± 0.02 Bc	2.46 ± 0.09 Ba	16.41 ± 1.42 Bb	21.71 ± 0.57 Ba
	P3	0.47 ± 0.07 Ab	2.17 ± 0.28 Bb	32.26 ± 6.39 Aa	15.12 ± 1.08 Bb
I2	P0	/	/	/	/
	P1	0.88 ± 0.09 Aa	2.30 ± 0.23 Ac	7.04 ± 1.21 Ab	9.42 ± 1.15 Ab
	P2	0.65 ± 0.02 Ab	3.07 ± 0.12 Ab	24.66 ± 2.4 Aa	31.57 ± 0.83 Aa
	P3	0.52 ± 0.11 Ac	4.00 ± 0.98 Aa	28.78 ± 6.27 Aa	21.30 ± 0.34 Ac
I		45.90 ***	22.08 **	0.14 ^ns^	573.18 ***
P		17.401 ***	8.31 **	44.68 **	1166.74 ***
I × P		7.34 **	4.66 ^ns^	3.84 ^ns^	26.09 ***

Note: Different lowercase letters (a, b, c, …) indicate significant differences in means among different phosphorus treatments under the same irrigation method (*p* < 0.05, n = 3); different uppercase letters (A, B, C, …) indicate significant differences in means among different irrigation treatments under the same phosphorus condition (*p* < 0.05, n = 3), assessed using one-way ANOVA and Duncan’s post hoc test. Data are presented as the mean ± standard deviation (SD) based on three replicates. The analysis employed two-way ANOVA, with ** and *** indicating significant differences at the *p* < 0.01 and *p* < 0.001 levels, respectively, and ^ns^ indicating no significant difference. PUE: phosphorus uptake efficiency; PFPP: partial factor productivity of phosphorus fertilizer; AUP: apparent utilization rate of phosphorus fertilizer; AEP: agronomic efficiency of phosphorus fertilizer; I: irrigation method; P: phosphorus application; I1: conventional irrigation; I2: micro-nano bubble water drip irrigation; P0: 0 kg·hm^−2^; P1: 86 kg·hm^−2^; P2: 172 kg·hm^−2^; P3: 258 kg·hm^−2^.

## Data Availability

The original contributions presented in the study are included in the article material; further inquiries can be directed to the corresponding authors.
